# Central Sensitization and Pain: Pathophysiologic and Clinical Insights

**DOI:** 10.2174/1570159X20666221012112725

**Published:** 2023-09-06

**Authors:** Michele Curatolo

**Affiliations:** 1 Department of Anesthesiology and Pain Medicine, University of Washington, Seattle, WA, USA;; 2 The University of Washington Clinical Learning, Evidence and Research (CLEAR), University of Washington, WAI, USA;; 3 Center for Sensory-Motor Interaction, University of Aalborg, Aalborg, Denmark;; 4 Center for Musculoskeletal Disorders, Harborview Injury Prevention and Research Center, University of Washington, Seattle, WA, USA

**Keywords:** Pain, central sensitization, pain mechanisms, pain therapy, diagnosis, translational research

## Abstract

Central sensitization is an increased responsiveness of nociceptive neurons in the central nervous system to their normal or subthreshold afferent input.

**Aim:**

To explain how the notion of central sensitization has changed our understanding of pain conditions, discuss how this knowledge can be used to improve the management of pain, and highlight knowledge gaps that future research needs to address.

**Methods:**

Overview of definitions, assessment methods, and clinical implications.

**Results:**

Human pain models, and functional and molecular imaging have provided converging evidence that central sensitization occurs and is clinically relevant. Measures to assess central sensitization in patients are available; however, their ability to discriminate sensitization of central from peripheral neurons is unclear. Treatments that attenuate central sensitization are available, but the limited understanding of molecular and functional mechanisms hampers the development of target-specific treatments. The origin of central sensitization in human pain conditions that are not associated with tissue damage remains unclear.

**Conclusion:**

The knowledge of central sensitization has revolutionized our neurobiological understanding of pain. Despite the limitations of clinical assessment in identifying central sensitization, it is appropriate to use the available tools to guide clinical decisions towards treatments that attenuate central sensitization. Future research that elucidates the causes, molecular and functional mechanisms of central sensitization would provide crucial progress towards the development of treatments that target specific mechanisms of central sensitization.

## 1 INTRODUCTION

Central sensitization is defined by the International Association for the Study of Pain (IASP) as an increased responsiveness of nociceptive neurons in the central nervous system to their normal or subthreshold afferent input [1]. Historically, the discovery that injury at peripheral tissues induces hyperexcitability of spinal cord nociceptive neurons [2] has prompted a massive research effort that has consistently confirmed enhanced responsiveness of central nociceptive pathways with different animal models [3]. Subsequently, human models of sensitization have been developed and applied to multiple pain conditions, demonstrating that human clinical pain is associated with altered indices of central sensitization [4].

The purpose of this review article is to explain how the notion of central sensitization has changed our understanding of pain conditions, discuss how this knowledge can be used to improve the management of pain and highlight knowledge gaps that future research needs to address.

## 2 FROM ANIMAL TO HUMAN RESEARCH

Pre-clinical research on central sensitization has been extensive and has elucidated molecular and functional mechanisms underlying enhanced central nociceptive processes with different animal models. A review of these mechanisms is outside the scope of this article and can be found in previous papers [5-10]. Human research is obviously limited in its ability to study mechanisms of central sensitization. Nevertheless, human pain models, functional and molecular imaging have provided converging evidence that central sensitization occurs and is clinically relevant to patients with different pain conditions.

### 2.1 Human Pain Models

Human pain models consist in applying a stimulus and recording a response related to pain. A recent systematic review has identified 269 studies using more than a dozen human models of sensitization [11]. A variety of stimulus modalities have been used, most commonly pressure, heat, cold, and electrical stimulation [12, 13]. Capsaicin (topical, intradermal, or intramuscular) [14, 15], intramuscular hypertonic saline [16, 17], and nerve growth factor (NGF) [18, 19] have been used to induce pain and hyperalgesia. While cutaneous pain has mostly been studied, models of muscle [18, 20-22] and visceral [23-25] pain have been developed and applied to human research. Methods to record responses related to pain include pain thresholds (the intensity of a stimulus that elicits pain), pain intensity after application of a standardized stimulus, area of pain, area of hyperalgesia, and tolerance time (time from application of a stimulus until the subject does not tolerate the pain) [12].

Temporal summation explores how ongoing or repeated peripheral signals are enhanced in the central nervous system, leading to the amplification of pain or pain with innocuous stimuli. Temporal summation is the human correlate of the wind-up phenomenon documented in animals, which is an increase in the activity of spinal nociceptive neurons during repeated stimuli of constant intensity [26]. Similarly, for temporal summation, repeated stimulation at constant intensity evokes an increase in pain perception [27] and an increase in the amplitude of the nociceptive withdrawal reflex [28]. Enhanced temporal summation has been detected in multiple pain conditions, and is considered a key mechanism of central sensitization [29, 30].

Endogenous inhibition of nociceptive processes is important in the homeostatic regulation of pain. Substantial pre-clinical research has elucidated mechanisms of endogenous inhibition, involving, among others, a top-down control by the endogenous supraspinal opioid system *via* descending noradrenergic and serotoninergic pathways [31]. Dysfunction of endogenous inhibition leads to pain amplification [32]. Endogenous pain inhibition is studied in humans by the conditioned pain modulation model [33]. Two painful stimuli are applied at two distant body areas: a “test” stimulus, and a “conditioning” stimulus. If endogenous inhibition is well functioning, the conditioning stimulus will reduce the pain response caused by the test stimulus. Dysfunction of conditioned pain modulation has a high prevalence in chronic pain [34] and is thought to be an important determinant of central sensitization and pain.

All the above models rely on self-report and are therefore subjective in nature. This is not necessarily a limitation, as pain is a subjective experience. Objective models have complemented the information obtained with self-report measures, and include the recording of spinal reflexes and cerebral evoked potentials. The nociceptive reflex is an electromyographic response of the lower limb following electrical stimulation, and has been used to study spinal nociceptive responses [35-38]. The use of brain evoked potentials has allowed the recordings of cerebral activity after painful stimulation [39-42].

With few exceptions, studies using human pain models have found that patients with different pain conditions display enhanced pain and nociceptive responses, compared with pain-free subjects. The findings have been summarized in a recent review [4].

### 2.2 Functional and Molecular Imaging

Neuroimaging has provided substantial progress in our understanding of the mechanisms of human pain. Overviews of neuroimaging studies in pain can be found in previous reviews [43-47].

In brief, functional and molecular imaging has been applied in conjunction with human pain models to understand cerebral mechanisms of augmented pain and nociceptive processes. Several studies that have used sensitization models in conjunction with functional magnetic resonance imaging (fMRI) have documented activation of pain-related brain areas, such as the primary somatosensory cortex, insula, anterior cingulate cortex, and prefrontal cortex [48-51].

Changes in brain chemistry have been revealed by magnetic resonance spectroscopy (MRS). Associations between enhanced pain responses and increased brain levels of the excitatory neurotransmitter glutamate have been detected in patients with fibromyalgia [52] and healthy volunteers [53], suggesting that enhanced glutamatergic signaling in brain-promoting areas is involved in central sensitization. Pain sensitivity has been shown to be negatively correlated with brain levels of the inhibitory neurotransmitter γ-aminobutyric acid (GABA) in patients with fibromyalgia [54] and healthy volunteers [53], suggesting that reduction in cerebral inhibitory processes is involved in central sensitization.

Neuroinflammation is associated with central sensitization in animal models [55-58]. Human studies using Positron Emission Tomography (PET) have detected neuroinflammation in the brain of patients with fibromyalgia [59] and chronic low back pain [60], and in the spinal cord/nerve roots of patients with radicular pain [61].

## 3 ARE WE MEASURING CENTRAL SENSITIZATION?

Human models have consistently shown enhanced responses in patients, compared with pain-free subjects [4]. The crucial question is whether these responses specifically reflect central sensitization. The IASP terminology for central sensitization specifies that changes in function have to occur in central neurons only, and peripheral neurons are functioning normally [1]. Increased responsiveness of peripheral neurons is the feature of peripheral sensitization, which is defined by the IASP as an increased responsiveness and reduced threshold of nociceptive neurons in the periphery to the stimulation of their receptive fields [1]. Peripheral sensitization is a key determinant of pain and hyperalgesia following peripheral events, such as trauma or inflammation [62-64]. The relationships between peripheral and central sensitization are illustrated in Fig. (**1**).

**Fig. (1):**
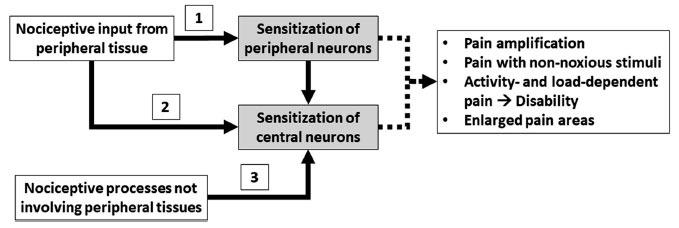
Nociceptive inputs from peripheral tissues can produce sensitization of peripheral neurons (peripheral sensitization) and sensitization of central neurons (central sensitization). Central sensitization can be induced also *via* sensitization of peripheral neurons (pathway 1). Central sensitization is observed also in pain conditions without evident pathology of peripheral tissues (pathway 3).

Thus, an increased pain response to peripheral stimulation in human models can be the result of peripheral sensitization, central sensitization, or both. For measures to be specific to central sensitization, they should be able to measure increased responsiveness in central neurons, while ruling out increased responsiveness of peripheral nociceptive neurons (pathways 2 and 3 of Fig. **1**). Human pain models cannot provide this information. The nociceptive withdrawal reflex mentioned above is thought to reflect spinal nociceptive activity, but strictly speaking also this model does not directly measure the responsiveness of nociceptive neurons.

When human pain models are applied to an area of injury, they cannot distinguish between peripheral and central sensitization, as the enhanced pain response can be the result of hyperactivity of both peripheral and central neurons. When however the stimulus is applied to a distant and healthy body site, one can argue that the increased pain response should be the result of central mechanisms [65]. For instance, for patients with neck pain after a cervical trauma, enhanced pain responses have been documented with stimulation of areas distant from the neck [66-68]. Because such areas are not injured and not painful, one can assume that the hypersensitivity is the result of enhanced responsiveness of central neurons.

Caveats apply to this assumption. A recent systematic review found evidence for raised inflammatory markers in patients with neck pain, and some markers were associated with clinical variables [69]. Circulating inflammatory mediators could sensitize peripheral nociceptors not only at the site of injury, but also at distant sites. Another study found signs of small fiber pathology, thermal hypoesthesia and hypersensitivity in neck pain [70], which can also cause generalized peripheral sensitization. Therefore, it is possible that peripheral mechanisms contribute to the hypersensitivity recorded after stimulation of non-injured and non-painful areas.

Overall, the current methods to assess central sensitization in humans are surrogate measures of uncertain validity. While they likely reflect, at least in part, central sensitization, their interpretation should be cautious.

## 4 NOCIPLASTIC PAIN

The IASP has recently introduced the term “nociplastic pain”: pain that arises from altered nociception despite no clear evidence of actual or threatened tissue damage causing the activation of peripheral nociceptors or evidence for disease or lesion of the somatosensory system causing the pain [1]. Clinical criteria to identify nociplastic pain have been proposed [71]. Some of them overlap with signs of central sensitization, but differences apply. The criteria for nociplastic pain require that the symptoms are not entirely explained by nociceptive or neuropathic mechanisms. According to the IASP, nociceptive pain arises from actual or threatened damage to non-neural tissue and is due to the activation of nociceptors; neuropathic pain is caused by a lesion or disease of the peripheral or central somatosensory nervous system. In clinical conditions, it is frequently challenging to determine that nociceptive and neuropathic mechanisms entirely explain the pain. In addition, the criteria for probable nociplastic pain include co-morbidities such as sensitivity to sounds, sleep disturbances, fatigue, and cognitive disorders. These characteristics are not typically part of the neurobiological processes involved in the determination of central sensitization, and not part of the definition of the IASP for central sensitization.

## 5 CLINICAL IMPLICATIONS

### 5.1 Assessing Central Sensitization

As outlined above, several surrogate methods to assess central sensitization in humans are available, but none of them is a validated diagnostic tool. In addition, most methods are time-consuming and some of them require expensive equipment, strongly limiting clinical applicability. Nevertheless, clinicians can use simple methods that can be embedded in clinical practice without significant time consumption and costs. These tests include the assessment of mechanical pain sensitivity using pressure algometers [72], allodynia to brush and cold [73], and pain areas using body maps [74]. Although a valid diagnosis of central sensitization is not feasible, findings of enhanced mechanical pain sensitivity outside the site of primary pain/injury, allodynia, wide referred pain areas, and multi-site pain are strongly suggestive of altered nociceptive/pain processes associated with central sensitization.

### 5.2 Explaining Pain

The notion that peripheral injuries of different natures induce enhanced nociceptive processes in the central nervous system has dramatically changed the way we explain pain. Before this knowledge was available, it was challenging to explain the frequent discrepancy between the magnitude of tissue damage and clinical manifestations. It is indeed common to observe severe pain and disability in patients with very limited evidence of peripheral lesions, or even without any detectable injury. While psychosocial components contribute to explaining pain with limited or no tissue damage, the notion of central sensitization has crucially improved our ability to provide patients with explanations for their condition. This is of great value to patients, who are as interested in having an explanation of their pain problem as they are in a cure or relief of their pain [75].

### 5.3 Treating Pain

Fig. (**2**) proposes a diagnostic and therapeutic pathway that makes use of indices of central sensitization to determine a treatment plan. This plan is supported by the current understanding of the pathophysiology of central sensitization, but warrants evaluation by clinical trials.

Patient education on the role of central sensitization should be considered part of the treatment. Understanding the role of central sensitization is of great importance to increase awareness of the multidimensional nature of pain, and can reduce the focus on tissue damage as the main or sole cause of pain. This can avoid perseverance in pursuing procedures or surgeries to remove a putative pain generator, when no such generator can be reliably identified. Accordingly, acceptance of treatments that act on the central nervous system, such as antidepressants, can be increased.

Both pharmacological and non-pharmacological treatments potentially reduce central sensitization. Among medications with central modulating action, the most widely used are antidepressants and anticonvulsants. Systematic reviews on antidepressants [76-80] and anticonvulsants [77, 81-83] have mostly demonstrated efficacy in different pain conditions, although the level of evidence was generally low and the effect size modest.

Opioids have an established role in the treatment of acute pain, but their benefits for chronic pain remain controversial, while there is evidence for harm [84, 85]. Opioids may induce hyperalgesia [86, 87], and can therefore enhance central sensitization. Based on the current knowledge, the use of opioids in patients with chronic pain and features of central sensitization does not seem appropriate.

Non-pharmacological treatments may produce pain relief also by reducing central sensitization. A recent meta-analysis found physical therapy to decrease temporal summation and enhance conditioned pain modulation [88]. Exercise can induce hypoalgesia, but can also exacerbate pain in patients with marked central sensitization [89]. While increasing physical activity may increase pain tolerance [90], there is some evidence that features of central sensitization, such as widespread mechanical and cold hyperalgesia, are associated with poor response to physical therapy [91]. The balance hypo-/hyperalgesia after exercise may depend on individual factors that are still to be clarified. Future research could address the question whether reducing central sensitization improves the efficacy of physical therapy. Psychological interventions can reduce pain [92]. Whether this effect is mediated by enhanced endogenous inhibition is an interesting matter of future research.

## 6 KNOWLEDGE GAPS

Despite substantial progress in the understanding of modulatory mechanisms leading to central sensitization, much work needs to be done to translate the current knowledge into benefits for patients. Crucial questions remain unanswered.

### 6.1 What Causes Central Sensitization?

When patients display enhanced pain responses after peripheral stimulation, we still do not know the cause. In analogy with animal studies, we assume that for patients with a documented peripheral pathology, such as osteoarthritis, the nociceptive input from the damaged tissue induces and maintains central sensitization. However, this does not explain central sensitization in many other clinical situations. For instance, what causes central sensitization when tissue pathology has apparently healed, such as after surgery or traumatic injury? What causes central sensitization when there has never been any documented source of peripheral nociception, such as in fibromyalgia (pathway 3 of Fig. **1**)? In such conditions, primary dysfunctions of central modulatory processes may be the determinants of central sensitization, but again, where do they come from?

The original models of central sensitization have been developed in animals by inducing peripheral tissue damage and recording central responses. This model does not seem to apply in many clinical pain conditions, where tissue damage is not documented or does not correlate with the clinical manifestations. Clinical pain is substantially different from experimental pre-clinical pain, and therefore warrants the development of different models to study central sensitization.

### 6.2 What are the Molecular and Functional Mechanisms of Central Sensitization?

Our current methods to study central sensitization in humans provide very limited insights into molecular and functional mechanisms. As mentioned above, functional and molecular imaging have provided progress. However, we have just started to scratch the surface. The mechanisms are likely to be different for different pain conditions and, within pain conditions, are likely different across patients. We still lack sensitive methods to understand mechanisms of central sensitization, and are even further away from being able to understand the pathophysiology at the individual level. This knowledge gap hampers the development of mechanisms-specific treatments.

## CONCLUSION

The knowledge of central sensitization has revolutionized the way we understand pain by providing a neurobiological explanation for common clinical conditions. Measures to assess central sensitization in patients are available. However, they lack validity in discriminating central from peripheral sensitization and, with few exceptions of demanding and costly procedures, do not provide information on functional and molecular mechanisms. Despite these limitations, clinical tools are available to identify central sensitization as a potential contributor to pain. Although the validity and diagnostic confidence are unknown and likely limited, it is appropriate to use the knowledge gained by clinical assessment to guide clinical decisions towards treatments that attenuate central sensitization. Future research that elucidates the causes, molecular and functional mechanisms of central sensitization would provide crucial progress towards the development of treatments that target specific mechanisms of central sensitization.

## Figures and Tables

**Fig. (2):**
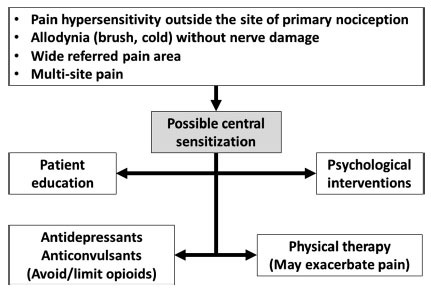
Proposed clinical assessment and treatment of central sensitization, based on current understanding of pathophysiology and available treatment modalities.

## LIST OF ABBREVIATIONS

fMRI: Functional Magnetic Resonance Imaging
IASP: International Association for the Study of Pain
NGF: Nerve Growth Factor
PET: Positron Emission Tomography
